# A Holistic Approach to Physiotherapy Treatment for Scheuermann Disease Along With Lumbar Canal Stenosis and Bilateral Lower Limb Radiculopathy: A Case Report

**DOI:** 10.7759/cureus.66194

**Published:** 2024-08-05

**Authors:** Pradhyum D Kolhe, Nikita G Zanwar, Pratik Phansopkar

**Affiliations:** 1 Musculoskeletal Physiotherapy, Ravi Nair Physiotherapy College, Datta Meghe Institute of Higher Education and Research (Deemed to be University), Wardha, IND; 2 Research and Development, Ravi Nair Physiotherapy College, Datta Meghe Institute of Higher Education and Research (Deemed to be University), Wardha, IND

**Keywords:** rehabilitation, physiotherapy, spinal injury, lumbar canal stenosis, scheurmann disease

## Abstract

Scheuermann disease, a structural deformity marked by kyphotic alterations in the thoracic or thoracolumbar spine, is frequently accompanied by back discomfort and spinal wedging. Scheuermann disease predominantly affects the thoracic and thoracolumbar parts of the spine; thus, there is an indirect link between the two conditions. This case report describes a rare form of Scheuermann illness in which lumbar canal stenosis caused bilateral lower limb radiculopathy.

A 50-year-old male with a confirmed diagnosis of Scheuermann illness complained of severe back discomfort, developing bilateral lower limb weakness, and sensory impairments. Clinical and radiographic investigations indicated lumbar canal stenosis at multiple levels, resulting in nerve root compression and radiculopathy. MRI reports of the lumbosacral spine were suggestive of severe, multiple-level degenerative changes. Spinal canal stenosis was noted at lumbar levels. Following the investigation findings suggestive of Scheuermann disease, the patient underwent posterior decompression and spinal fixation of L3-L4 and L4-L5. The outcome led to the decompression of the lumbar canal and the stabilization of the affected spinal segments. A personalized postoperative rehabilitation plan was developed based on the patient's complaints of pain, stiffness, and the difficulties he faced associated with the disease. Overall physiotherapy rehabilitation plays an essential role in the overall care of Scheuermann's illness and postoperative lumbar spine disease, aiding in functional restoration, enhancing quality of life, and encouraging long-term spinal health. Although exercise therapy is intense, it shows promising results and is beneficial for these conditions. Physical therapy in the postoperative period plays a significant role in promoting the patient's functional independence.

## Introduction

Scheuermann disease is often referred to as Scheuermann osteochondrosis of the spine or juvenile kyphosis of the spine [1]. It is diagnosed by anterior wedging of more than or equal to 5 degrees in three or more contiguous vertebral bodies [2]. Pathological abnormalities found in the endplates and discs of the spine are associated with certain radiological characteristics, including irregular endplates and disc lesions [3]. Scheuermann disease predominantly affects the thoracic and thoracolumbar parts of the spine, thus there is an indirect link between the two conditions. Lumbar canal stenosis is a condition that is becoming more prevalent in the elderly population. In the United States, there are over 200,000 individuals suffering from lumbar spinal stenosis causing significant pain and dysfunction [4]. Each year, an increasing number of people diagnosed with lumbar canal stenosis go through symptoms such as discomfort in the lower back pain or numbness in the lower extremities and intermittent claudication caused by nerve issues [5]. The most prevalent symptoms are lower back pain and a loss of sensation in the lower limbs [6]. Magnetic resonance imaging is significant because it can detect the severity of lumbar canal stenosis without requiring any invasive treatments. Spinal canal stenosis has been shown to produce nerve root compression. Although decompressive surgery is beneficial, older individuals benefit less than younger patients and they are typically omitted from studies examining postoperative physiotherapy [7]. In this case report, we aim to showcase a holistic approach to physiotherapy treatment for a patient with Scheuermann disease secondary to lumbar canal stenosis and bilateral lower limb radiculopathy. By detailing the patient’s treatment plan and outcomes, we seek to emphasize the effectiveness of a comprehensive physiotherapy strategy in addressing both the underlying condition and the associated symptoms.

## Case presentation

Patient information

A 50-year-old male, farmer by occupation, reached out to the Physiotherapy department with symptoms of lower back discomfort for the previous two years as well as trouble completing everyday duties. The pain was insidious in onset, gradual in progression, and mild in intensity initially but increased to moderate in the last three months. The pain was aggravating on movement and while doing farm activities; it radiated to both the lower limbs and was associated with a tingling sensation in both lower limbs. It was relieved on rest and with analgesic medications. The patient had consulted at a private hospital for the above complaint where an MRI-lumbosacral spine was done. Table 1 shows the timeline of events.

**Table 1:** Timeline of events

Date of Admission	9/10/2023
Date of Surgery	15/10/2023
Date of Physiotherapeutic Rehabilitation	17/10/2023
Date of Discharge	20/1/2024

On examination, the patient was seen in the supine position with the head supported by a pillow, upper limbs by the side, and lower limbs relaxed and extended. The patient was mesomorphic with no complications after the surgery. Patient assessment details taken before the rehabilitation included manual muscle testing (MMT), range of motion (ROM), pain assessment by the visual analog scale (VAS), along with Oswestry Disability Index scores and functional independence measure (FIM). Pre-rehabilitation manual muscle testing is shown in Table 2 and the pre-rehabilitation range of motion is described in Table 3.

**Table 2:** Pre-rehabilitation MMT 0: No visible or palpable contraction; 1 (Trace): visible or palpable contraction (no range of motion); 2- (Poor-): partial range of motion, gravity eliminated; 2 (Poor): full range of motion, gravity eliminated; 2+ (Poor+): gravity eliminated/slight resistance or less than half range against gravity; 3- (Fair-): more than half but less than the full range of motion, against gravity; 3 (Fair): full range of motion against gravity; 3+ (Fair+): full range of motion against gravity, slight resistance; 4- (Good-): full range of motion against gravity, mild resistance; 4 (Good): Full range of motion against gravity, moderate resistance; 4+ (Good+): full range of motion against gravity, almost full resistance; 5 (Normal): normal, maximal resistance MMT: manual muscle testing

S.No.	Muscle group	Right	Left
1	Hip flexor muscles	2	2
2	Hip extensor muscles	2	2
3	Hip abductor muscles	2	2
4	Hip adductor muscles	2	2
4	Knee flexor muscles	4	4
5	Knee extensor muscles	4	4
6	Ankle plantarflexion muscles	5	5
7	Ankle dorsiflexion muscles	5	5

**Table 3:** Pre-rehabilitation range of motion

Side of joint	Right	Right	Left	Left
Type of Movement	Active	Passive	Active	Passive
Hip Flexion	0^0^-10^0^	0^0^-15^0^	0^0^-10^0^	0^0^-15^0^
Hip Extension	10^0^-0^0^	15^0^-0^0^	10^0^-0^0^	15^0^-0^0^
Hip Abduction	0^0^-30^0^	0^0^-35^0^	0^0^-35^0^	0^0^-40^0^
Hip Adduction	0^0^-30^0^	0^0^-30^0^	0^0^-30^0^	0^0^-30^0^
Knee Flexion	0^0^-100^0^	0^0^-105^0^	0^0^-100^0^	0^0^-105^0^
Knee Extension	0^0^-100^0^	105^0^-0^0^	0^0^-100^0^	105^0^-0^0^

Radiological imaging findings

**Figure 1:**
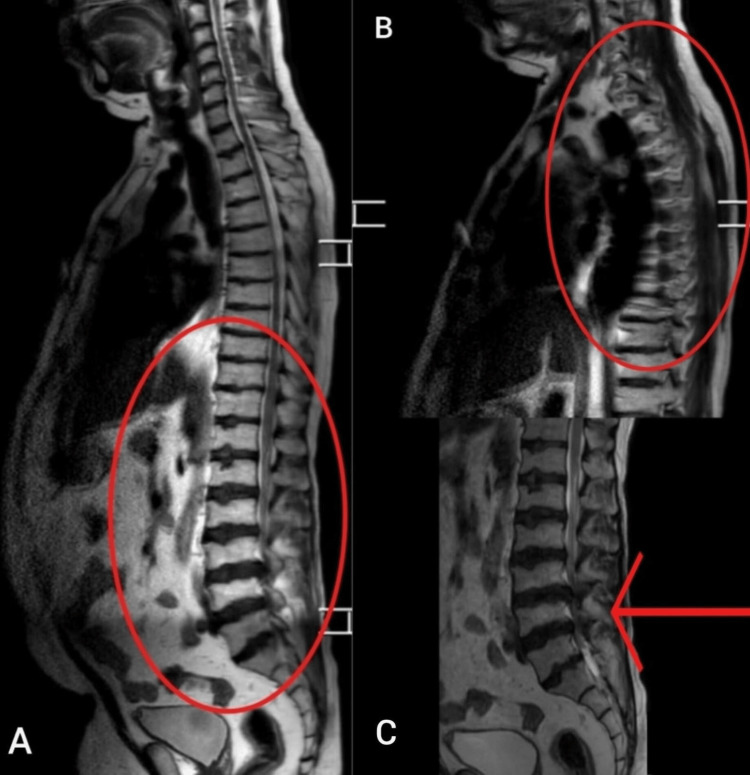
MRI of the lumbosacral spine (lateral view) MRI: magnetic resonance imaging

Investigations using an MRI of the lumbosacral spine (lateral view) suggest kyphosis with multiple osteophytes seen at various levels (Figure 1A and Figure 1B). This was suggestive of severe, multiple-level, degenerative changes. Spinal canal stenosis at the lumbar level was also noted (Figure 1C). The findings were suggestive of Scheuermann disease. Demographic information on Scheuermann disease features involves vertebral level and their numbers, amount of degenerative disc disease, and associated magnetic resonance imaging findings of patients. Following the diagnosis, posterior decompression and spinal fixation of L3-L4 and L4-L5 were recommended. No post-surgical complications were seen. The patient was conservatively managed by thoracic bracing.

Physiotherapy rehabilitation

A single physical therapist devised and delivered personalized face-to-face physiotherapy to avoid any inter-rater disagreements. Physiotherapy involved a weekly five-session adaptive program organized into four phases to treat particular deficiencies and restore independent standing and ambulatory abilities. Rehabilitation was divided into four phases over a 12-week duration as shown in Table 4.

**Table 4:** Duration of rehabilitation

Phase	Stages	Duration of rehabilitation
1	Extremity and core muscle Training	1 week
2	Balance and stance training	1 month
3	Gait training	8 weeks
4	Sit-to-stand training	12 weeks

The duration of each phase was decided by the performance of the preceding phase's obligations. The first phase concentrated on strengthening the extremities and core muscles to prepare for motor function [8]. Therapy began one week following surgery and continued throughout the program. Workouts began in a supine posture and progressed to a standing stationary position. In the supine position, isometric exercises were performed for the lower extremities [9]. Dynamic, active, assisted exercises for upper extremities were also performed [10]. The physiotherapy exercise protocol followed during rehabilitation is shown in Table 5 [7].

**Table 5:** Physiotherapy exercise protocol

Exercise	Description	Goal
Bilateral ankle pumps	The patient is asked to raise their legs while lying in bed and move their ankles back and forth.	10 repetitions of 1 set
Hip abduction and adduction	The patient is positioned on their side and instructed to move their hips outwards and then inwards. The sides are then switched.	10 repetitions of 1 set
Heel slides	The patient is asked to extend their legs completely and bring heels toward their buttocks comfortably.	10 repetitions of 1 set
Sit to stand on a walker	The therapist assists the patient in standing with the support of a walker.	1-2 times with progression in repetition
Bilateral heel raises	The patient is instructed to lift their heels while sitting on the walker.	10 repetitions of 1 set
Bilateral knee extension/flexion	The patient is asked to flex and extend their knees while seated on the walker.	10 repetitions of 1 set
Restorator	The patient is instructed to rotate the upper extremity strengthening machine using their arms.	10 repetitions with 3 rounds
Ball-squeeze exercise	The patient is instructed to place a rubber ball between their legs and then flex and relax against the resistance provided by the ball.	10 repetitions per 3 sets
Isometric quadriceps contraction	The patient initially began without resistance and later advanced to using 2.5 lbs ankle weights for isometric quad squeezes.	5 for 10 seconds for each contraction
Scapular mobility exercise	The patient is asked to perform isometric contraction and relaxation of the scapular muscles.	10 for 5 seconds for each contraction
Bilateral marching	With assistance, the patient is asked to march in place to work on hip flexors and later with a resistance weight cuff on the ankle.	10 repetitions with 1 set
Electrical muscle stimulation	The patient is asked to enhance muscle activation - paraspinal muscles, abdominal muscles, gluteal muscles, quadriceps, and hamstring muscles.	7-10 minutes per session

During the second phase, aquatic equilibrium and stance training were implemented to adapt to the upright posture. Surgical incisions had healed sufficiently for safe water exposure during the second phase, which began around a month following surgery. Aquatic therapy was recommended over terrestrial training because it reduced axial stress on the spine [11]. The focus of the second phase was on promoting standing and movement. Each day, a session was conducted for a period of five weeks. The primary objective was to enhance the duration of standing while minimizing the need for assistance. Patients in the third phase underwent gait training to prepare for the physical demands of walking. Gait training consists of tandem walking, walking sideways, using the parallel bar, and treadmill training. This enhances muscle memory, which improves walking [12]. The patient has to be able to stand for at least 10 seconds, either with or without help. The fourth phase is based on sit-to-stance training [13].

Outcome measures 

Outcomes were measured pre- and post-rehabilitation by the physical therapist. The visual analog scale score while exercising decreased from 6/10 to 2/10 following rehabilitation [14]. Furthermore, Oswestry Disability Index scores were compared before and after rehabilitation [15]. A clinical study of Oswestry Disability Index data demonstrates a good impact of the rehabilitation program. The patient's strength was evaluated before surgery and at the end of the fourth phase by evaluating the muscles related to their lumbar and sacral nerve roots. Strength was tested using the Medical Research Council Scale for Muscle Strength, which ranges from 0 to 5+and the results showed an improvement (Table 6) [16]. Furthermore, the range of motion of the hip joint was recorded using goniometry, and improvements were observed in post-rehabilitation (Table 7) [17]. Functional independence measures properly analyze a person's limitations in self-care, sphincter control, mobility, and locomotion. This was used to assess each patient before surgery and at the end of the fourth phase of rehabilitation [18]. Table 8 shows the outcome measure scales in evaluation metrics.

**Table 6:** Post-rehabilitation manual muscle testing 0: No visible or palpable contraction; 1 (Trace): visible or palpable contraction (no range of motion); 2- (Poor-): partial range of motion, gravity eliminated; 2 (Poor): full range of motion, gravity eliminated; 2+ (Poor+): gravity eliminated/slight resistance or less than half range against gravity; 3- (Fair-): more than half but less than the full range of motion, against gravity; 3 (Fair): full range of motion against gravity; 3+ (Fair+): full range of motion against gravity, slight resistance; 4- (Good-): full range of motion against gravity, mild resistance; 4 (Good): full range of motion against gravity, moderate resistance; 4+ (Good+): full range of motion against gravity, almost full resistance; 5 (Normal): normal, maximal resistance

S.No.	Muscle group	Right	Left
1	Hip flexor muscles	4+	4+
2	Hip extensor muscles	4+	4+
3	Hip abductor muscles	4+	4+
4	Hip adductor muscles	4+	4+
4	Knee flexor muscles	5	5
5	Knee extensor muscles	5	5
6	Ankle plantarflexion muscles	5	5
7	Ankle dorsiflexion muscles	5	5

**Table 7:** Post-rehabilitation range of motion

Side of joint	Right	Right	Left	Left
Type of Movement	Active	Passive	Active	Passive
Hip Flexion	0^0-^110^0^	0^0-^120^0^	0^0-^110^0^	0^0-^120^0^
Hip Abduction	0^0-^35^0^	0^0-^45^0^	0^0-^35^0^	0^0-^45^0^
Hip Adduction	0^0^-40^0^	0^0^-45^0^	0^0^-40^0^	0^0^-45^0^
Knee Flexion	0^0^-100^0^	0^0^-105^0^	0^0^-100^0^	0^0^-105^0^
Knee Extension	0^0^-100^0^	105^0^-0^0^	0^0^-100^0^	105^0^-0^0^

**Table 8:** Evaluation metrics VAS: visual analogue scale, FIM: functional independence measure

S.No.	Scale	Pre-rehabilitation	Post-rehabilitation
1	VAS	6 score	2 score
2	Oswestry Disability Index scores	40	10
3	Functional independence measure (FIM)	38	12

## Discussion

Scheuermann disease is often referred to as Scheuermann osteochondrosis of the spine. The condition is characterized by the endplate of the growth cartilage being affected by a disease that is believed to be primarily caused by repeated strain on the growth cartilage. This strain is further exacerbated by an inherited susceptibility [19]. The mentioned prevalence in the general population was between 1% and 10% [20]. Scheuermann disease predominantly affects the thoracic and thoracolumbar parts of the spine, thus there is an indirect link between the two conditions. Approximately 200,000 individuals in the United States experience lumbar spinal stenosis, which refers to a narrowing of the spinal canal at the lumbar region. It is connected with a reduction in accessible space for the lumbar spine's neuronal and vascular structures. Standing, walking, or lumbar extension frequently worsens the disease, whereas forward flexion, sitting, or recumbency relieves it [4]. In their case report, Jain et al. mention Scheuermann disease in adolescents in India is associated with a positive family history. They concluded that while not all cases necessitate immediate surgical intervention, there may come a point where surgery becomes necessary. In the interim, the administration of medications and participation in physiotherapy are crucial components of the treatment process. The case presented here was Scheuermann disease secondary to lumbar spinal stenosis. Initially, the patient was managed conservatively by thoracic bracing and later managed by physical therapy. A comprehensive rehabilitation plan was developed in phases for the postoperative period. Scheuermann disease treatment consists of rehabilitation, bracing, and in rare cases, surgery. Physiotherapy regimens without Scheurmann's disease (isolated lumbar canal stenosis) typically focus on pain relief, improving flexibility, strengthening the muscles around the spine, and enhancing overall functional mobility. Pain management by applying heat or cold packs to the affected area reduces pain and inflammation. Techniques like transcutaneous electrical nerve stimulation can help in pain relief. Gently stretching the hamstrings relieves tension in the lower back. Exercises like bridges, planks, and abdominal contractions strengthen the muscles that support the spine. Sports that involve exerting significant pressure on the spine like weightlifting as well as those that subject the vertebra to repetitive stress, such as rugby, should always be avoided [19].

## Conclusions

This case study highlights the valuable outcomes of postoperative rehabilitation for those with Scheuermann disease and lumbar canal stenosis. Despite encouraging discoveries, there is still much to investigate and learn in this field. Collaboration among physiotherapists, orthopedic surgeons, and other healthcare professionals from different disciplines is crucial for creating personalized treatment plans and ensuring a smooth continuum of care. Education and guidance on correct body mechanics, posture, and adjusting activities are essential elements of rehabilitation. This empowers patients to actively engage in their recovery and maximize long-term results. More studies on rehabilitation for Scheuermann disease patients with lumbar canal stenosis can lead to significant breakthroughs and improvements in treatment methods. Overall physiotherapy rehabilitation plays an essential role in the overall care of Scheuermann's illness and postoperative lumbar spine disease, aiding in functional restoration, enhancing quality of life, and encouraging long-term spinal health.
